# Sexually transmitted diseases and HIV risk behavior in adolescents compared with adults in Arad county

**DOI:** 10.1186/1471-2334-13-S1-P85

**Published:** 2013-12-16

**Authors:** Laura Nicolescu, Dana Negru, Cristina Cotuna, Cristiana Cioroboiu

**Affiliations:** 1 Public Health Department, Arad, Romania

## Background

Young people are exposed to numerous risk behaviors that can affect their present life and perspectives as adults. Commonly risk behaviors for them are: regular smoking, more than 5 glasses of alcohol in the last month, attempted suicide in the past year, marijuana use in the past month, consumption of other illicit drugs in the last year and unprotected sex at any time of life, resulting in unwanted pregnancy and school dropout. Sexually transmitted diseases (STD) and HIV are also involved as major consequences.

## Methods

A comparative study of STD and HIV risk behavior was made to determine the prevalence of condom use, illicit drugs, alcohol and smoking, in Arad, for two age groups 14-19 and over 20 years. Data were obtained from 759 anonymous questionnaires and were organized in Statistical Package for Social Sciences 14.0. The observed frequencies, where differences arose significance, were assessed using p-value by Chi square test for comparing groups, and Fischer test.

## Results

People under 20 correctly answered in 80% when asked whether a healthy looking person can be HIV infected, compared to the 72.7% adults which answered correctly (p<0.012). Teens were also more aware of HIV transmission route, compared to adults (p<0.027). Young people knew other STDs in 84.8%, adults only at the rate of 72.4%. (p<0.000). Even so, only 9.4% of adolescents had had an HIV test compared to 41.0% of adults (p<0.000). Adolescents always used, in 29.6%, condoms with casual sex partners compared to 14.2%, in adults (p<0.000). Sexual partners were HIV tested in 8.7% of cases compared to 31.4% for adults (p<0.012).

## Conclusion

Even though teens theoretically know more STDs and they are using condoms more frequently with casual sex partners, HIV testing for them or their partners is not a common practice.

